# The applications of artificial intelligence in liver transplantation: A comprehensive scoping review

**DOI:** 10.1371/journal.pone.0361120

**Published:** 2026-10-23

**Authors:** Aditya Borakati, Joshua Agilinko, Jack Wu, Samora Hunter, Anwar Alhaq, Farid Froghi, James Teo, William Bernal, Chris Callaghan, Miriam Cortes-Cerisuelo, David Wallace

**Affiliations:** 1 Roger Williams Institute of Liver Studies, King’s College London, London, United Kingdom; 2 Department of Liver Transplantation and Hepatopancreatobiliary Surgery, King’s College Hospital, London, United Kingdom; 3 Department of Endocrine Surgery, Hammersmith Hospital, Imperial College Healthcare NHS Trust, London, United Kingdom; 4 School of Cardiovascular & Metabolic Medicine and Sciences, King’s College London, London, United Kingdom; 5 CogStack, Precision Medicine, Networked Services, King’s College Hospital NHS Foundation Trust, London, United Kingdom; 6 Department of Hepatopancreatobiliary Surgery, Hammersmith Hospital, Imperial College Healthcare NHS Trust, London, United Kingdom; 7 Liver Intensive Therapy Unit, King’s College Hospital, London, United Kingdom; 8 Department of Renal Transplantation, Guy’s and St Thomas’ NHS Foundation Trust, London, United Kingdom; 9 Department of Hepatopancreatobiliary Surgery and Liver Transplantation, Royal Free Hospital, Royal Free London NHS Foundation Trust, London, United Kingdom; 10 London School of Hygiene and Tropical Medicine, London, United Kingdom; Hospital Universitario La Paz, SPAIN

## Abstract

Liver transplantation (LT) is a complex surgical intervention, with intricate decision-making and risks at all stages before, during and after the procedure itself. Artificial intelligence (AI) may assist the process but has seen limited use. This scoping review assessed primary articles reporting on clinical AI in LT in MEDLINE and Embase from 2000 to September 2026. 196 articles were identified: focusing on the pre-operative period before transplant (21.9%), organ retrieval (9.7%), intra-operative issues (3.6%), short-term post-operative complications (16.8%), long-term post-operative complications (45.9%) and natural language processing (2.0%). 95.9% had retrospective designs and only 12.8% had direct multi-centre data, with the rest from national registries or single centres. 13.8% of studies externally validated their AI tools. Only one study performed a prospective evaluation of their tool with a randomized controlled trial. Progress towards clinically deployable tools will require harmonised, higher-granularity datasets, consistent reporting, and prospective evaluation within real-world transplant workflows.

## Introduction

Liver transplantation is a highly effective treatment for end-stage liver disease, but it is complex and carries significant risks before, during, and after the procedure. The decision to offer a transplant involves careful consideration of patient wishes, surgical fitness, prognosis, graft availability, and, in some countries, resource and financial constraints. While some livers are donated by living individuals, most grafts in the Western World are recovered from deceased donors following brainstem (DBD) or circulatory death (DCD) [1]. Unfortunately, the demand for transplants far exceeds supply, and in the USA, pretransplant mortality remains 13.4 deaths per 100 patient-years, with 12.4% of candidates removed from the liver waiting list in 2024 having died or become too sick for transplant [2].

Artificial intelligence (AI) may help address some of these challenges by supporting risk prediction and decision making. For example, to allocate these scarce organs, most countries use risk scores—such as MELD or UKELD—to prioritise patients based on predicted mortality without a transplant [3], though alternative approaches considering post-transplant survival are also debated, with the UK being the first to implement an allocation system based on overall Transplant Benefit [4]. At the time of organ retrieval, surgeons assess graft suitability based on donor status, graft appearance, and recipient condition, but there are no standardised protocols for these decisions. During surgery, choices regarding implantation technique and anatomical variations can further complicate the process. After transplantation, patients face acute complications such as haemorrhage, vascular issues, and rejection, as well as long-term risks including graft failure, infection, malignancy, renal failure, and cardiovascular disease, all of which are exacerbated by immunosuppressive therapy. In each stage of the transplantation process, AI has the potential to standardize many of the aforementioned issues including through more accurate waitlisting, graft assessment and prediction of post-operative outcomes.

The stages in the liver transplantation process, along some of the long-term complications, are outlined in Fig 1.

**Fig 1:**
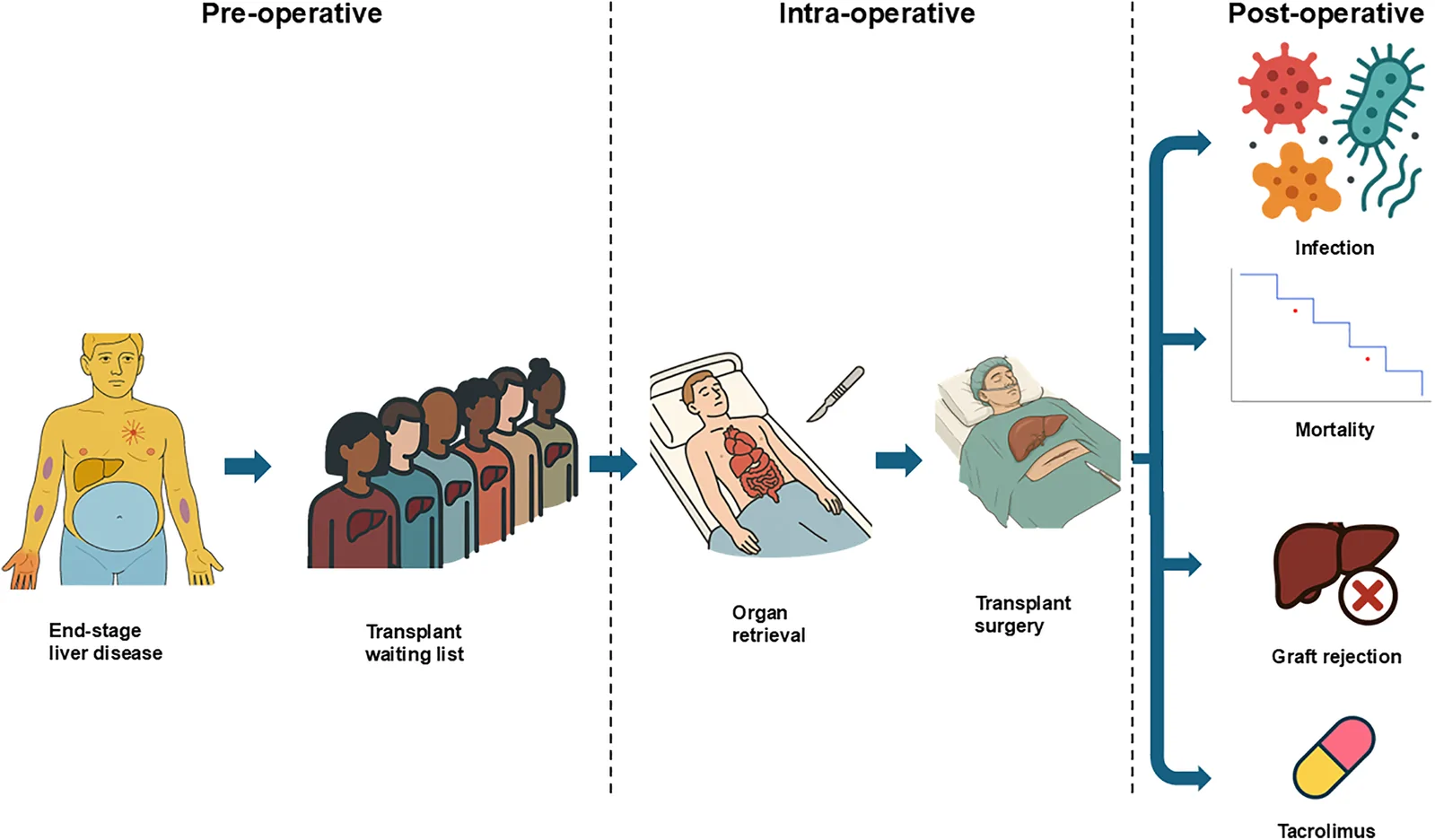
Stages of the Liver Transplantation process with examples of post-operative complications (some graphics in this figure were generated by generative AI software: Microsoft Copilot, Microsoft, Redmond, WA, USA, the authors grant permission for publication of this figure under a Creative Commons Attribution License (CCAL) CC BY 4.0).

### Background to artificial intelligence and its evolution

Over the last decade, and notably since 2022, the popularity of transformer-based large language models such as ChatGPT has resulted in a surge in artificial intelligence (AI) applications. AI has the potential to transform healthcare, by assisting clinical decisions [5], automation of tasks, and improving efficiency. Complex multifactorial clinical activities such as liver transplantation are therefore well placed to benefit from AI.

AI and machine learning (ML) are often used interchangeably and have varying definitions in the literature. In this review, we define AI as an umbrella term referring to any tool designed to perform tasks that would otherwise require human intelligence. ML is considered a subset of AI involving algorithms that discover patterns and improve performance on a task through exposure to data [6].

To rigorously evaluate these technologies, however, a clear methodological stratification is required, tracking a chronological evolution from transparent statistical models to complex, “black-box” deep learning frameworks.

Historically, **classical statistical models** and **rule-based expert systems** formed the earliest foundations of clinical AI. Rule-based systems rely on hardcoded, human-defined logical paths (e.g., “if-then” clinical algorithms) to automate decision-making. As data availability expanded, these gave way to data-driven, statistical approaches like logistic regression and Cox proportional hazards regression. Within this tier, advanced automated regularization techniques—such as **LASSO (Least Absolute Shrinkage and Selection Operator)**—are frequently classified as machine learning because they autonomously handle variable selection and model shrinkage to optimize predictive power without manual human curation.

While structurally distinct from true AI, these standard approaches are frequently evaluated alongside **traditional, task-specific machine learning (ML) classifiers**. This tier includes models like decision trees, random forests, support vector machines (SVM), and gradient boosting architectures (e.g., XGBoost, CatBoost). These models are typically reported in the medical literature to handle structured datasets, predicting outcomes either as discrete categories (classification) or a range of continuous numbers (regression) [7].

A major evolutionary milestone occurred with the advent of **deep learning (DL)**. Powered by artificial neural networks (ANNs) and convolutional neural networks (CNNs), these models are inspired by biological networks with complex, multi-layered, interconnected inputs and outputs [8].

The current state-of-the-art is driven by **transformer models** and **natural language processing (NLP)**. Built on deep neural networks, transformers possess the unique ability to “focus attention” on important segments of text while identifying context across entire datasets [9]. When scaled, these transformers undergo **generative pre-training (GPT)**, an autonomous learning process where the model learns the underlying structure of language by predicting the next word in vast textual repositories (such as the entire public internet). These foundational steps have created **large language models (LLMs)** which power popular ‘chatbot’ style AI such as ChatGPT [10]. These highly advanced models can bridge multiple domains, including generating clinical narratives, reading unstructured health records, image analysis, and advanced prediction modelling.

As clinical modelling has evolved along this spectrum, a critical trade-off has emerged between predictive performance and model interpretability. While classical regression models offer direct, intuitive coefficients (such as odds or hazard ratios), advanced ML and deep learning algorithms operate as “black boxes” with hidden non-linear layers that prevent clinicians from deducing why a specific prediction was made. In high-stakes environments like organ allocation or post-operative risk tracking, this lack of transparency severely hinders clinical trust. Consequently, the paradigm of Explainable AI (XAI) has emerged, using post-hoc frameworks like Shapley Additive Explanations (SHAP) to assign quantitative importance to predictor variables [11].

Understanding this methodological spectrum and the necessity of interpretability frameworks is essential to accurately map the literature. The purpose of this paper is to evaluate the state of the art of AI applications across this stratified taxonomy at each stage of the liver transplantation process and identify current evidence gaps in the literature.

## Methods

We conducted a scoping review of the literature, searching for all published articles on the use of artificial intelligence in applications related to liver transplantation. The review was conducted in accordance with the Joanna Briggs Institute guidelines on scoping reviews [12–14] and reported using the PRISMA-ScR checklist [15] (S1 File). The protocol for the scoping review was pre-registered on the Open Science Framework: https://osf.io/tuvrz/ [16].

### Eligibility criteria

All primary research articles evaluating artificial intelligence tools on patients eligible for, undergoing or those who have undergone a liver transplant were included. There was no age restriction on the patient subjects. Pre-prints, in addition to published, peer-reviewed articles, were eligible for inclusion.

Review articles, correspondence, and editorials were not eligible for inclusion. Non-English language articles were also excluded.

### Search strategy

MEDLINE and Embase were searched from 2000 to September 2026, using a combination of Medical Subject Headings and free-text terms related to liver transplantation and artificial intelligence (S2 File). The latter database also incorporates pre-print articles from databases such as arXiv and medrXiV and these were also included.

Title and abstract screening was performed on the Rayyan platform (rayyan.ai [17]), by two independent reviewers (AB and JA). Any disagreements were resolved through consensus or discussion with a third reviewer (DW).

### Data items and charting process

One reviewer (AB) reviewed full-text publications and extracted relevant data to a pre-piloted spreadsheet in Microsoft Excel for Windows.

Data extracted included study title, first author, year, country of origin of the study population, timing on the transplant process (pre-transplant, organ retrieval, intraoperative, post-operative complications) and details about the AI model if available, including the type of model.

Additional information recorded included study design (retrospective vs. prospective), data source (single-centre/ multi-centre/ registry) and validation approach for the model (internal only or on external datasets).

A formal risk of bias assessment was not conducted due to the heterogeneity of studies included and these are not required in a scoping review as per PRISMA-ScR guidelines [15].

### Synthesis of results

Eligible papers are presented through a narrative synthesis. Included papers were partitioned into the different stages of the perioperative transplant pathway, i.e., pre-transplant, organ retrieval, intraoperative, post-operative complications and the purpose of the AI tool. Each paper was assigned to a single stage; papers whose main focus was a natural language processing method were charted as a separate category. Evidence gaps were identified through discussion between the review team and summarised in text, as well as a table.

### Bibliometric analysis

We quantified the number of included publications by year in a bar chart and generated a world map, showing the number of publications by country.

In addition we conducted a simple bibliometric analysis using the Bibliometrix software [18], with default settings.

## Results

### Search results

We identified 2,193 records through our database search, of which 300 were duplicates. After title and abstract screening of the remaining 1,893 records, we selected 466 records for full-text screening. This identified 196 articles that met our search criteria and were included in the final review (Fig 2). Bibliographic details of all included articles are available in the S3 file.

**Fig 2:**
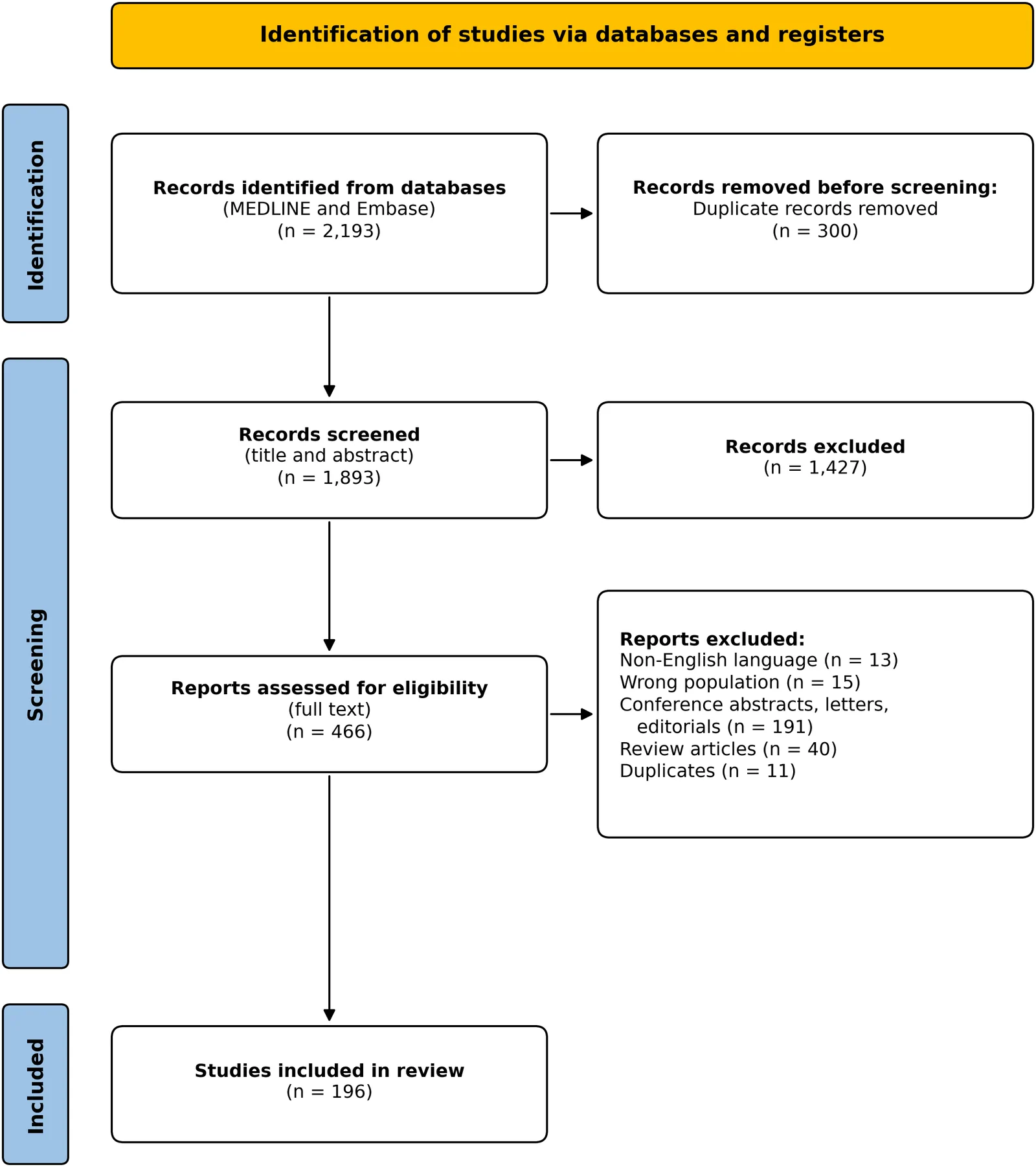
PRISMA flowchart of inclusion and exclusion of records.

163 (83.2%) articles were published from 2020 onwards, from 2020 there is a year-on-year rise in publications to 2025; 2026 includes publications to September only (Fig 3). The largest number of studies used datasets from the United States (n = 72, 36.7%), followed by China (n = 36, 18.4%). The United Kingdom produced the sixth most studies (n = 9, 4.6%). Studies using data from more than one country were counted once for each country. Fig 4 displays a map with the frequencies of included articles per country.

**Fig 3:**
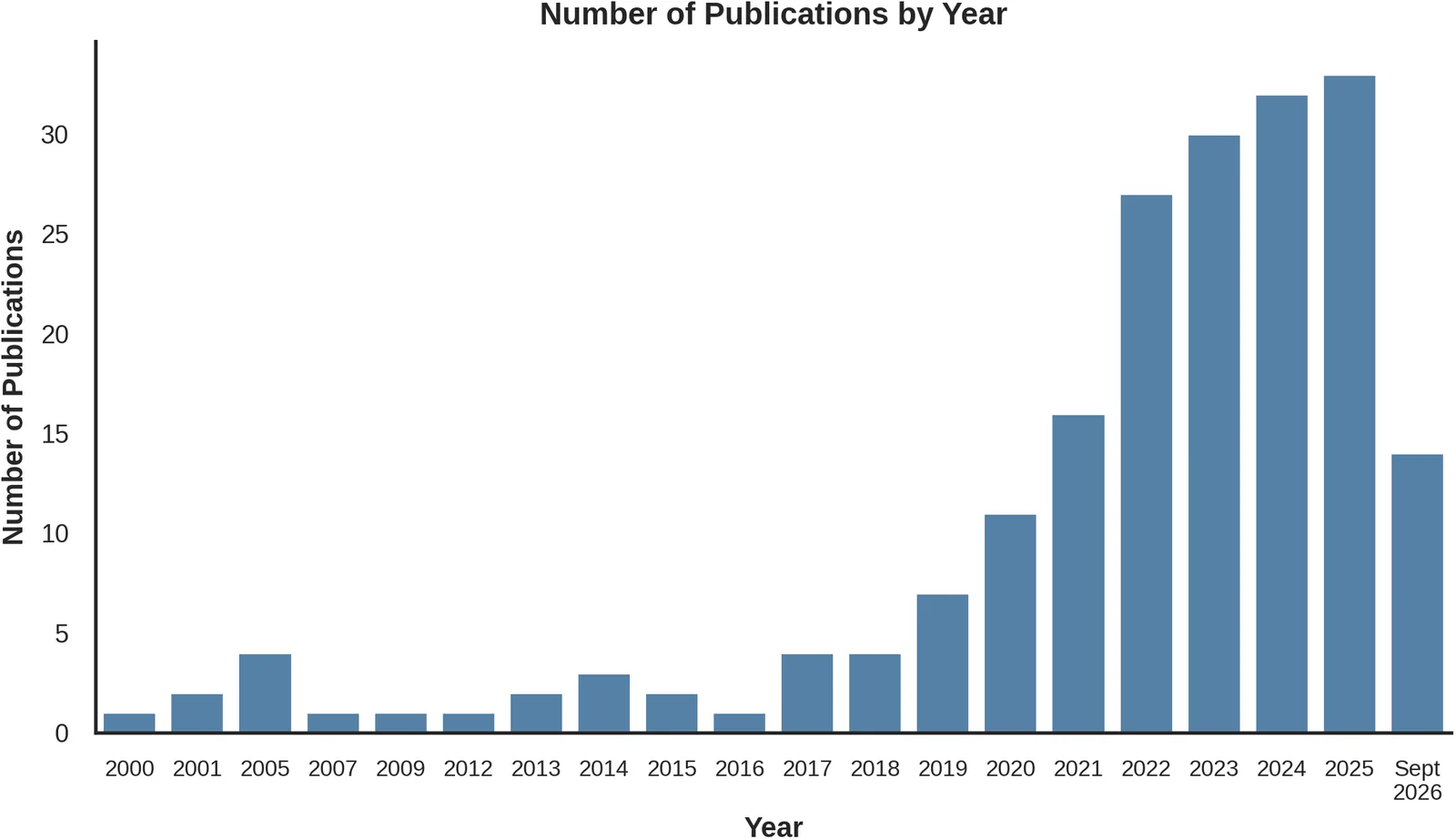
Number of publications on AI in liver transplantation by year of publication.

**Fig 4:**
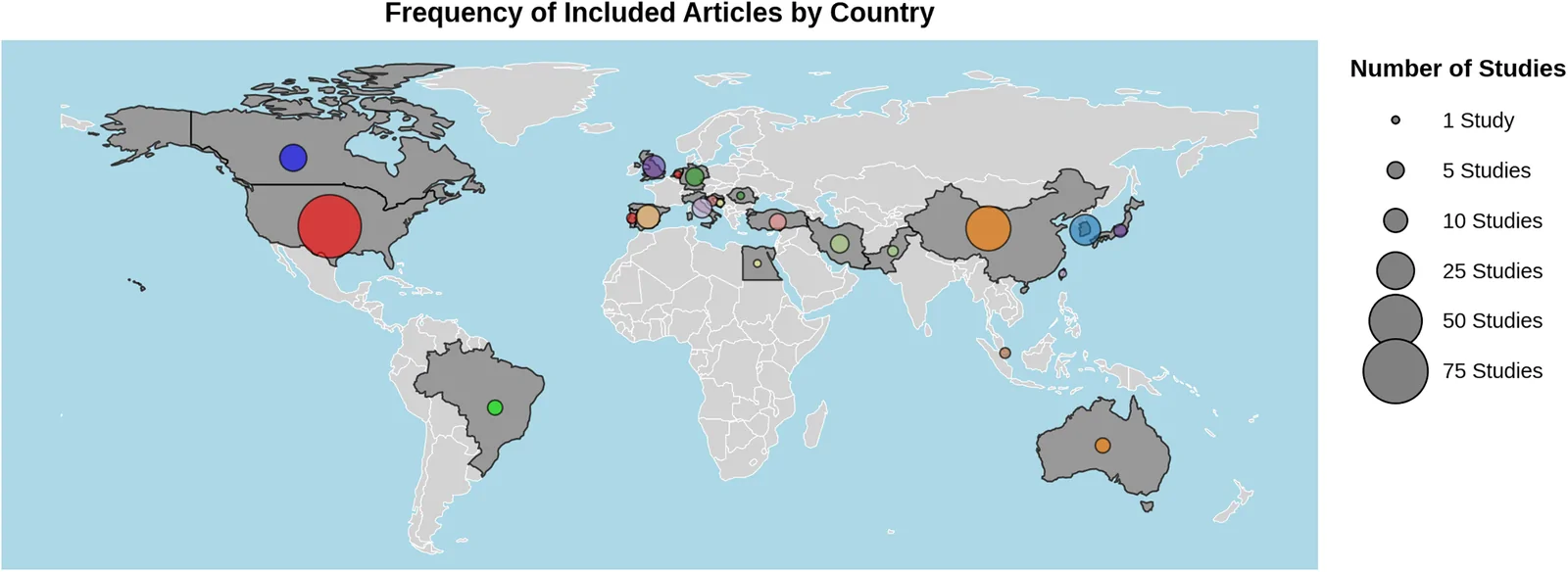
World map showing distribution and frequency of included articles by country (figure generated in Python using Plotly; two studies using multinational registry data are not shown. The authors grant permission for publication of this figure under a Creative Commons Attribution License (CCAL) CC BY 4.0).

### Bibliometric analysis

In addition to the above, a bibliometric analysis of the 177 included articles indexed in Scopus (19 were not indexed and could not be analysed) is given in S4 File. This analysis notably highlights the contribution of individual institutions such as the University of Toronto, the University Health Network and the Mayo Clinic, which appeared 24, 22 and 18 times respectively in author affiliations. The most locally cited article, cited by 20 of the other included articles, was by Lau et al [19].

### Study design

188 (95.9%) studies were conducted retrospectively. 25 (12.8%) studies had data from multiple centres, while 60 (30.6%) used national registry data and the remainder were single-centre. Only 27 (13.8%) studies externally validated their models on populations unrelated to their training data.

### Overall themes

The overall themes charted in the scoping review are shown in Fig 5.

**Fig 5:**
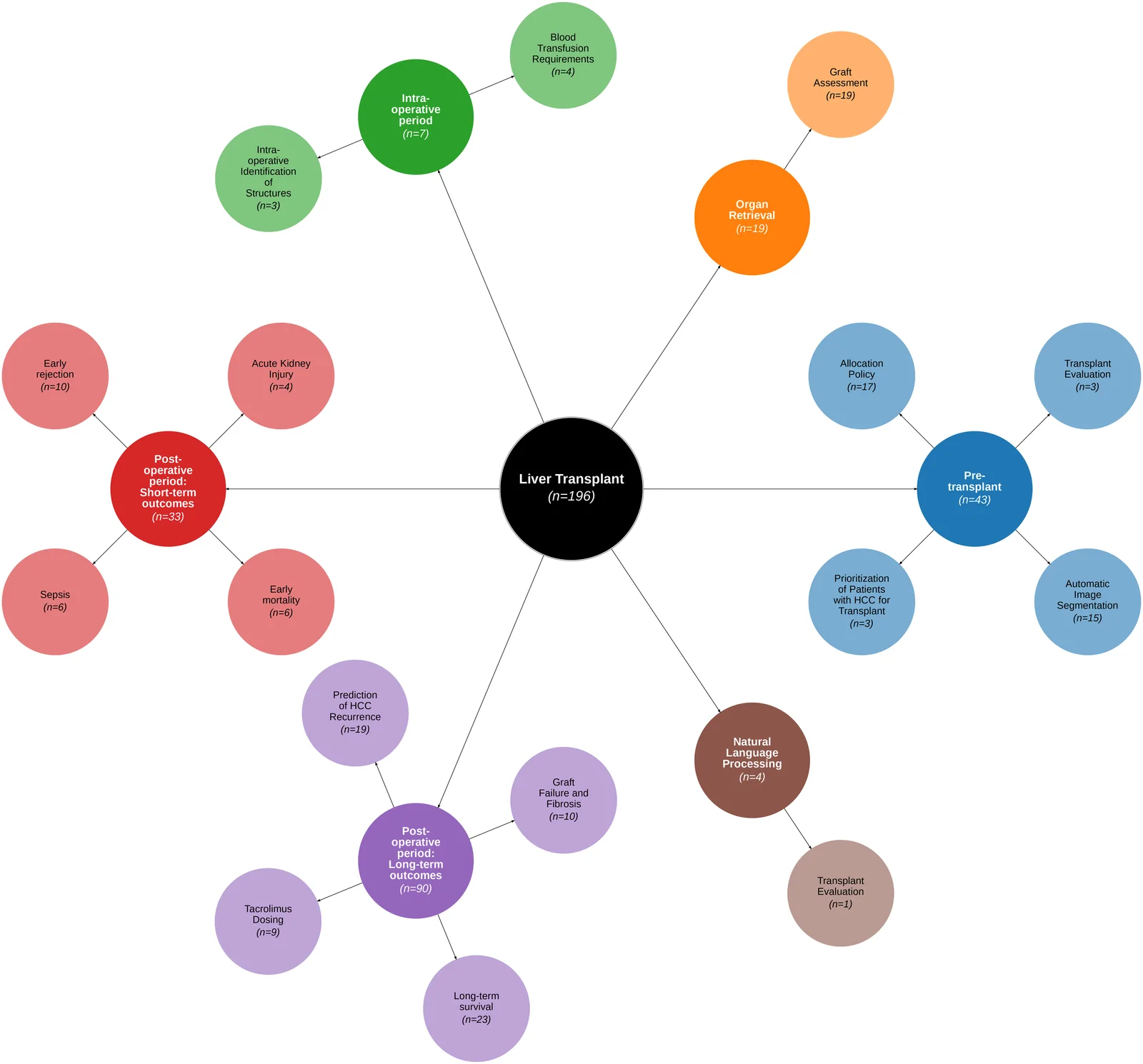
Network diagram of themes charted in review; HCC: Hepatocellular Carcinoma. The Transplant Evaluation node under Natural Language Processing shows the one NLP study that also addressed transplant evaluation.

43 studies (21.9%), focused on the pre-transplant period. 19 articles (9.7%) focused on the organ retrieval process. These were all studies that aimed to assess the quality of the donor graft using AI techniques. 7 articles (3.6%) studied issues intra-operatively, these included predicting intra-operative transfusion requirements and identifying anatomical structures intra-operatively using computer vision. 33 articles (16.8%) studied short-term post-operative outcomes, within 90-days of transplant including mortality, graft failure, acute kidney injury and sepsis. 90 articles (45.9%) explored complications in the long-term post-operatively. These were predominantly complications such as mortality or graft loss, but also included tumour recurrence for transplants performed for oncologic purposes (pre-dominantly hepatocellular carcinoma). NB- Not all studies map to one of these themes so are not counted.

A further key theme is the use of machine learning models for prediction of tacrolimus dosing. There were also four further articles (2.0%) that assessed natural language processing (NLP) methods, most often to extract concepts from electronic health records (EHRs) on datasets including liver transplant recipients [20–22].

## Pre-transplant

### Transplant assessment

Four studies assessed the role of AI in assessing potential recipients for their suitability for a transplant. Strauss and colleagues performed a qualitative study evaluating patients’ opinions of a hypothetical AI clinical decision support tool for listing a patient for transplant. While there was some optimism for the potential of AI to improve fairness and reduce discrimination in listing, there was concern that AI may not be trustworthy and may not act in the patient’s best interests [23].

Hasjim et al. [24] (preprint) went one step further and used a large language model to simulate four participants in a transplant selection committee: surgeon, hepatologist, cardiologist and social worker. This simulation was able to accurately identify contra-indications to transplant in 98.4% of cases and predicted 6-month mortality with 94.9% accuracy. However, there were significant errors with this model as it failed to identify significant prognostic factors such as a lack of social support or severe cardiorespiratory disease.

Zaver [25] and co-workers identified AI enhanced electrocardiograms (ECGs) as an adjunct to the transplant pre-assessment, where patients are assessed for fitness for an elective liver transplant. Using a neural network on ECG tracings, they were then able to predict post-transplant atrial fibrillation and left sided heart failure, although with weak AUCs of 0.69 for each. A fourth study, by Robitschek et al. [22], used a large language model to quantify the effect of psychosocial risk factors and social determinants of health on listing decisions, and is described in the natural language processing section.

### Allocation policy

17 articles applied machine learning methods to prioritise patients for liver transplant. Several aimed to improve upon the MELD score, which is widely used for waiting list prioritisation and especially in the US. For example, Cooper [26] (preprint), Cucchetti [27], and Salkic [28] developed neural network models that showed modest to significant improvements in predicting 90-day mortality compared to MELD, with improvements in area under the receiver operating curve (AUC) ranging from 0.05 to 0.16. Some models [26] also reported increased fairness in allocation, with women and patients with primary sclerosing cholangitis prioritized compared to MELD. However, these studies were limited by small sample sizes or lack of external validation, raising concerns about overfitting (where the model predicts the training dataset too closely and cannot be generalised to other data) and reproducibility. More recent single-centre studies from Iran reported similar results. Aghazadeh et al. [29] found support vector machine and naïve Bayes models predicted waiting list mortality with AUCs of 0.85 and 0.88, respectively, whilst the LASSO-derived ‘Sina score’ of Hosseini-Asl et al. [30], which added hand grip strength and skeletal muscle mass to routine blood results, achieved an AUC of 0.75 for 3-month mortality compared with 0.61 for MELD. Both cohorts were small, with only 9 deaths among 193 candidates in the former and 64 patients in the latter, and neither was externally validated.

Ahn et al. [31] supplemented traditional biochemistry-based models with ECG analysis, improving mortality prediction when combined with the adapted allocation model MELD-Na. Gomez-Orellana et al. [32] used a neural network trained on the UK transplant registry and externally validated it in Australia, demonstrating higher concordance with 90-day mortality than MELD-based models. Older models using decision trees, such as Bertisimas [33], also outperformed MELD-Na and, in simulation, could reduce deaths and gender imbalance.

Norman [34] and Kwong [35] evaluated machine learning models for waiting list dropout in hepatocellular carcinoma (HCC) patients, but found traditional Cox regression performed comparably and was preferred for its simplicity and explainability. Halimi et al. [36] combined a LightGBM model with SHAP-based supervised clustering to predict mortality in candidates with HCC, outperforming MELD-based and tumour scores with only eight variables and identifying seven subgroups of increasing risk, although again without external validation. In paediatric liver disease, Kulkarni [37] and Rajanayagam [38] used machine learning to identify risk factors for poor outcomes and develop risk scores for these outcomes respectively. Serum creatinine and ventilatory support were identified as key predictors in the former study, while the latter study’s model achieved an excellent accuracy metric of 0.96 AUC. This, however, was limited by an extremely small sample size of 54 patients and no external validation, meaning this figure is likely overfitted and unlikely to generalize.

Donor-recipient matching has also been explored as a method of allocating organs. This is where factors from both donor and recipient are used to compute the predicted benefit of the transplant match and used to prioritize allocation [39]. Haydon [40] and Briceno [41] applied neural networks to match donors and recipients, achieving higher predictive accuracy than MELD-based or other Cox regression models, though model complexity and lack of explainability limit clinical adoption. Further studies found logistic regression sometimes outperformed machine learning [42], and logistic regression remained preferred for allocation decisions due to its simpler interpretability [43].

### Image segmentation

For specific types of liver transplantation, such as living donor, paediatric and transplantation for cancer, accurate pre-operative planning with imaging is essential. This is traditionally carried out manually, by radiologists to identify regions of interests, including margins of lesions, the volume of the liver (volumetry), and identifying relevant vascular and biliary structures [44].

There were 15 articles identified which used AI to automate this process. These articles made use of both CT [45,46] and MRI [47] imaging and were able to accurately identify volumetry [45,48–50], biliary structures [47] and porto-systemic venous vasculature [45,49]. Madbouly et al. confirmed the accuracy of such models in determining volumetry on live donors by comparing these estimates with the resected graft [51]. Mun and colleagues [48] went further and obtained sequential images of living donors before and after hepatectomy and trained a model to predict regeneration, which may allow prediction of safe resection volumes. Most recent studies used convolutional neural networks, typically variants of the U-Net model, whereas earlier studies used knowledge-based, classifier-based or semi-automatic methods. Twelve of the 15 studies used single-centre data and the remaining three used registry data; only one study externally validated its model, again limiting generalisability.

## Organ retrieval

Nineteen articles assessed the quality of donor grafts during the retrieval process. Most livers transplanted worldwide are from deceased donors, whose graft quality is typically assessed visually by surgeons for steatosis (fat content of the liver), cirrhosis (advanced scarring of the liver), or malignancy. Grafts from living donors are typically healthier and assessed pre-operatively for function, however in the context of deceased donors, surprise pathologies can present during retrieval surgery. While machine perfusion devices can provide quantitative assessment, their use is limited by cost and complexity. Thus, there is ongoing need for objective, scalable methods to assess graft quality, and several studies have explored AI-based image and biochemical analysis for this purpose.

The earliest, by Kim et al. [52] in 2005, used ultrasound images and liver function tests from living donors to predict graft steatosis with a neural network, using biopsy as the reference standard. Their model achieved an AUC of 0.97, outperforming radiologists (AUC 0.91), though the study was again limited by small sample size and lack of external validation.

One early approach used smartphone images of discarded and accepted grafts to train a support vector machine classifier, achieving an AUC of 0.77, with sensitivity and specificity of 95% and 81% respectively, using biopsy as the reference standard [53]. Piella et al. [54,55] developed a similar model and mobile application using a random forest classifier, trained on 192 donors with standardised imaging and additional blood results. This model achieved an AUC of 0.82 and overall accuracy of 86% for predicting steatosis, outperforming surgical visual assessment (73% accuracy). More recently, Amara et al. [56] automated steatosis assessment from pre-retrieval CT in 147 deceased donors; a two-step model combining the liver-spleen attenuation difference with radiomic features achieved an AUC of 0.87 for macrovesicular steatosis of 30% or more, although all scans came from a single organ procurement organisation.

Other studies focused on biochemistry and donor characteristics. Pettit et al. [57] used the UNOS registry to identify factors associated with successful liver or kidney donation in deceased donors. Their random forest classifier achieved an AUC of 0.91 and specificity of 87%, with donor weight, age, and liver function tests as the most influential variables. However, the UNOS dataset only includes donors who donated at least one organ, introducing a bias towards higher positive predictive value. Frey et al. [58] used the SRTR dataset to predict significant macrosteatosis (>30%) with a lasso regression model (AUC 0.71). Pontes-Balanza [59] applied XGBoost to create a model to select grafts for discard or transplantation, based on macroscopic descriptions of the graft and clinical parameters from the donor. They achieved an AUC of 0.79 for predicting untransplantable grafts but consistent with many other studies, the model was limited by a small sample size and single centre study design.

Zhixing et al. [60] predicted delayed graft function in recipients using XGBoost on 5,242 donor-recipient pairs in China, achieving an AUC of 0.88 in training/validation and 0.78 on external data. Key predictors included shorter perfusion time, higher donor sodium, gamma glutamyl-transferase, and glucose. However, this model also incorporated operative and recipient factors, limiting its use for graft-only assessment.

Several other articles assessed biopsy samples from donor grafts [61–68], aiming to improve consistency and turnaround time. Gambella et al. [61] reported an intraclass correlation coefficient of 0.93 between their convolutional neural network and pathologist reports for steatosis identification. Kue et al. [69] applied an AI model to preimplantation frozen sections from 161 grafts; AI-quantified steatosis was associated with early allograft dysfunction (OR 1.63) but not with graft or patient survival.

The studies reported here were done either before the era of machine perfusion (techniques to preserve or improve the quality of liver grafts) or without comparison to machine perfusion metrics. These devices are now ubiquitous in Western transplant centres and in most cases allow objective measurement of liver function leading to an increased utilization of more suboptimal grafts [70]. Future studies of donor graft assessment tools should incorporate the impact of machine perfusion and their functional assessment.

## Intra-operative period

### Blood transfusion requirements

Four studies have explored the use of AI to predict blood transfusion needs during liver transplantation [71–74]. Given the complexity of the procedure and the frequent presence of coagulopathy or varices in patients, significant blood loss is common, necessitating transfusion.

Liu et al. [71] developed an XGBoost model using data from 1,193 transplant recipients, identifying portal hypertension, hepatitis, liver function, and pre-operative haemoglobin as the strongest predictors. The model achieved an AUC of 0.81 and 72% accuracy in training and validation, with external validation showing 76% accuracy. While promising, the practical impact may be limited in settings where blood products are routinely available, but such models could be valuable in resource-constrained environments.

Chen et al. [72] conducted a similar study in China, focusing on massive transfusion (defined as 18 units of red blood cells). Their CatBoost model, trained on 1,239 patients and externally validated on 46, achieved an AUC of 0.81, with 68% overall accuracy, 89% sensitivity, and 66% specificity. Key predictors included pre-operative haemoglobin and activated partial thromboplastin time.

Park et al. [73] evaluated neural networks and logistic regression in a Korean cohort of 414 patients. While neural networks showed a slightly higher AUC (0.851 vs 0.840), logistic regression was preferred for its simplicity and explainability. Wakiya et al. [74] (preprint) also used logistic regression, with feature selection repeated across 100 random data splits, to predict massive blood loss (80 mL/kg or more) in 290 transplants at a single Japanese centre; the final 11-variable model achieved an AUC of 0.74 on test data.

### Intra-operative identification of anatomical structures

Three studies assessed AI for real-time identification of anatomical structures during pure laparoscopic living donor hepatectomy, all from the same Korean group. Using indocyanine green injection to highlight bile ducts, Oh et al. [75] annotated 30 intraoperative videos and applied the DeepLabV3 + model. The model achieved Dice coefficients of 0.73 for biliary structures and 0.43 for transection sites compared to manual expert annotation. Although results were modest and based on a small cohort, this approach shows potential for assisting surgeons and reducing the risk of problematic post-operative bile leaks. The group subsequently predicted the bile duct bifurcation site, with 93.3% clinical accuracy on an independent test set but a Dice coefficient of only 0.47 [76], and segmented vascular structures and the avascular dissection plane for right liver mobilisation in 48 videos from three institutions, with Dice coefficients of about 0.65 on external validation [77].

## Post-operative period: Short-term outcomes

There were 33 included papers which assessed AI for predicting short-term post-operative outcomes within the first 90 days of transplant. These explored a range of outcomes, including acute kidney injury (AKI), sepsis, acute rejection and cardiovascular events.

### Acute Kidney Injury

Machine learning models have shown promise in predicting post-operative AKI, which has significant consequences on long term prognosis in liver transplant [78]. Gradient boosting (AUC 0.75) identified pre-operative bilirubin, low intra-operative urine output, and long anaesthesia time as key predictors [79]. Random forest models in donors after cardiac death (DCD) achieved higher accuracy (AUC 0.85), with bilirubin and prolonged anhepatic phase as significant factors [80]. Neural networks using variables such as MELD score, pre-existing kidney disease, and intra-operative haemorrhage outperformed logistic regression (AUC 0.81 vs 0.71), though again these studies were limited by small sample size and lack of external validation [81]. Other gradient boosting models (AUC 0.76) reinforced the importance of pre-operative bilirubin and intra-operative factors [82].

### Sepsis and infection

AI models have also been applied to predict sepsis and infection. An XGBoost model using physiological data achieved an AUC of 0.97, with sensitivity and specificity over 90% [83]. Random forest classifiers predicted sepsis within 7 days post-transplant (AUC 0.75), outperforming the SOFA score (AUC 0.64) [84]. XGBoost models for pneumonia prediction (AUC 0.73) identified routine blood results and operation time as key predictors [85]. Freire et al. [86] used a random forest to predict carbapenemase-resistant enterobacteria prior to transplant, achieving an AUC of 0.83, sensitivity of 66%, and specificity of 83%. The latter study was the largest in this domain, with 1544 patients included across 2 hospitals in Brazil and Italy. The generalisability of this study is still limited however, until true external validation is performed. The remainder of the studies were single centre, albeit with medium sized cohorts, with the largest containing 677 transplant patients. A further limitation is the complexity of some of these models, with Kamaleswaran’s model being developed from 311 features. In a two-centre Chinese study, Cao et al. [87] developed stacking ensemble models for early intra-abdominal infection (n = 363), with AUCs of 0.80 and 0.86 on external validation for the pre- and post-operative models.

### Early graft dysfunction and rejection

Neural networks have been used to predict early rejection, within 90 days of transplant, with reported AUCs up to 0.95 and high sensitivity and specificity of 94% [88,89], though both studies lacked external validation. Lau et al. [19] used a random forest classifier to predict early graft failure at 30 days (AUC 0.82) which outperformed both the MELD score and the US Donor Risk Index [90]. Deep learning applied to ultrasound and clinical data (AUC 0.97) outperformed radiologists in assessing graft failure, and improved diagnostic accuracy for both senior and junior clinicians [91]. All studies were performed in single centre cohorts, with no external validation limiting generalisability to other patient populations. Further the latter study only had 6 radiologists from the same centre acquiring the ultrasounds, which is known to be extremely operator dependent [92], raising the question whether this can generalise to other operators in addition to other patient populations. More recently, Sun et al. [93] used a support vector machine to predict the perioperative co-occurrence of T-cell-mediated rejection and pneumonia in 717 recipients (test set AUC 0.79). Ding et al. [94] (preprint) used a multi-agent framework, in which large language models direct machine learning pipelines, to identify rejection biomarkers in the multicentre CTOT-14 trial dataset, reporting an AUC of 0.88 for liver graft rejection.

### Early mortality

Six studies assessed AI models for predicting mortality within 90 days of transplant. The largest, using national registry data from Canada, the UK, and the US, found ridge logistic regression outperformed other machine learning models (AUC up to 0.70) [95]. Molinari’s simple points-based neural network models (AUC 0.95) were highly explainable and correlated well with observed mortality [96], while Ershoff’s more complex neural network model (AUC 0.71) lacked transparency and had poor sensitivity [97]. Simpler neural networks with fewer predictors had lower accuracy (AUC 0.64) [98]. Random forest models in single-centre Asian cohorts achieved high AUCs (0.94 and 0.899) [99,100]. Cox regression and random forest models identified hyperlactaemia, MELD score, and organ failure as key predictors of mortality in Cheong’s study [101].

### Miscellaneous

Additional studies addressed outcomes such as pressure injury in children (AUC 0.84) [102], major cardiovascular events at 30 days (AUC 0.84 [103]), and acute graft-versus-host disease (AUC 0.86 [104]). Logistic regression and random forests were used to identify patients likely to benefit from enhanced recovery after surgery pathways (AUC 0.76), with risk scores based on MELDNa, intra-operative transfusion, and surgery duration [105]. XGBoost models predicted harmful alcohol use post-transplant for alcoholic liver disease (AUC 0.93 in training, specificity 90.8%, sensitivity 57.3%; AUC 0.692 and specificity 95.6% on external validation [106]). The latter study was conducted with patients from 10 different centres, however, the overall cohort was only 116 patients and only 34 (29%) had post-operative harmful alcohol use. The drop in AUC in the external validation set suggests extreme overfitting. Other recent models predicted deep vein thrombosis within 7 days, where a logistic regression nomogram (AUC 0.81 in a hold-out set of only 32 patients) was retained and machine learning comparators were regarded as exploratory [107], and acute respiratory distress syndrome, where a random forest (AUC 0.77 internally and 0.84 on external validation in 115 patients) outperformed the lung injury prediction score [108].

## Post-operative period: Long-term outcomes

There were 90 studies which used AI techniques to predict long-term outcomes after transplant. The most common focus was overall recipient survival and/or survival of the graft (the latter being distinct from the former as patients may be re-transplanted). However, specific subgroups included predicting recurrence of hepatocellular carcinoma after transplant, predicting fibrosis following transplantation, and optimising tacrolimus dosing.

### Long-term survival

Twenty-three studies concentrated on predicting overall survival after transplantation. Notably:

Nitski et al. [109] developed a transformer-based model using the US SRTR dataset, externally validated in Canada, to predict 1- and 5-year survival. The model achieved an AUC of 0.807 at 1 year and 0.722 at 5 years, with donor/recipient age, pre-operative MELD score, and serum sodium as key predictors.

Yu et al. [110] evaluated multiple machine learning models for 1-year survival on a multicentre Korean dataset, finding that a random forest classifier outperformed Cox regression, MELD, and BAR, with an AUC of 0.77. Key predictors included cold ischaemia time, recipient weight, and age. Kantidakis [111] conducted a large study using the UNOS registry (62,294 patients), where the random forest classifier (C-index 0.622) performed only marginally better than Cox regression (C-index 0.620). Recent registry studies reported similarly modest gains. Pettit et al. [112] found XGBoost outperformed reconstructed SRTR regression models for 1-year liver graft survival (AUC 0.71 vs 0.62) in UNOS data, and Waller et al. [113] used random survival forests in 3,774 retransplant recipients, with 1-year survival of 63.7% in the high-risk tertile compared with 88.7% in the low-risk tertile. De Oliveira et al. [114] found discrimination fell below an AUC of 0.70 on temporal validation in a Brazilian state registry, with logistic regression giving the most consistent performance.

### Graft failure and fibrosis

There were 10 papers evaluating long-term graft loss and graft fibrosis.

Raji [115] was among the first to use a neural network to predict graft survival after liver transplantation over 12 years, training a 3-layer model on 384 patients from the UNOS dataset. Theu achieved an AUC of 0.99, 100% specificity, and 99% sensitivity, but the small sample size and lack of censoring again suggest overfitting. Etayo et al. [116] also used a neural network to identify risk factors for 1-year graft loss in a Spanish cohort of 596 patients, finding donor and recipient age most significant, though no overall accuracy metrics were reported.

Azhie [117] and Qazi [118] focused on predicting graft fibrosis, a marker of recurrent liver disease and graft loss. Azhie [117] trained a long short-term memory neural network on 1,893 UNOS patients, using clinical and blood test data to predict fibrosis confirmed by biopsy, achieving an AUC of 0.798, similar to transient elastography. Qazi [118] used features from post-operative CT scans in a Canadian cohort of 254 patients, combining annotated imaging and clinical parameters to achieve an AUC of 0.811. Yu et al. [119] developed a U-Net model to automate reporting of post-operative biopsies, achieving an F-1 score of 0.89 and 89% accuracy compared to manual reporting. Koivu et al. [120] added transient elastography to clinical data in an XGBoost model, achieving an AUC of 0.90 for significant graft fibrosis in 197 recipients. Sharma et al. [121] developed GraftIQ, a neural network that combines clinician-derived probabilities with clinical and laboratory data to classify the cause of graft injury; trained on 5,217 biopsies, it achieved an AUC of 0.90 and was externally validated in the US, Germany and Singapore. Yoshimura et al. [122] predicted graft loss within 180 days in 748 living donor recipients and stratified patients into early, intermediate and late graft loss groups.

### Tacrolimus dosing

There were 9 studies using machine learning models to find optimal dosing for tacrolimus, which is the most commonly used immunosuppressive medication in solid organ transplantation [123]. Tacrolimus has a narrow therapeutic index, with a range of side effects including renal dysfunction, tremors and psychiatric complications, while subtherapeutic doses may lead to rejection and graft loss. Hence, optimal dosing is paramount in this setting.

Khong and colleagues [124] conducted a phase 2 randomized controlled trial (n = 62) comparing standard of care tacrolimus dosing to their ‘PPM’ algorithm [125]. The PPM model significantly reduced deviations from the target range (p = 0.029) and shortened hospital stay by 33% without increasing adverse events.

Retrospective studies by Woillard et al. (XGBoost) [126], Ponthier [127], and Li et al. (MARS, XGBoost [128]) demonstrated that machine learning models could predict inter-dose AUC with only two concentration measurements. Yoon et al. [129] successfully validated a long-short term memory neural network that maintained a median performance error of 8.8% in an external cohort. Huo et al. [130] trained a long short-term memory network on 1,597 kidney and liver recipients to predict next-day trough concentrations in hospital, with a mean absolute error of 1.88 ng/mL.

Song [131] and Tan [132] developed models for dosing in paediatric liver transplantation, with Song’s ridge regression model showing the lowest deviation in infants and Tan’s adaptation of Khong’s model achieving a low mean prediction error of −0.10. Song’s study had only 163 patients and Tan’s only 16, the former is still very large for a study in this rare patient cohort, however, given paediatric transplantation’s high clinical heterogeneity, it may be unlikely these models will generalize despite the successful replication in Tan’s study.

### Hepatocellular carcinoma (HCC) recurrence and mortality

Nineteen studies evaluated long-term outcomes in liver transplant recipients with HCC, and in particular focused on overall survival and post-transplant HCC recurrence:

Tran et al. [133] developed and externally validated the RELAPSE score (random forest classifier) on nearly 5,000 US and 941 European patients, achieving C-statistics of 0.81 and 0.77, respectively, in predicting post-transplant recurrence. Key risk factors included pre-transplant alpha-fetoprotein, neutrophil-lymphocyte ratio, tumour size, and vascular invasion. Neural network approaches by Ivanics [134] and Nam [135] showed similar predictive performance but lacked external validation.

Alternative methods for predicting recurrence included radiomics and genomics: Guo et al. [136] used LASSO Cox regression on pre-transplant CT features (C-index 0.79), while Liu et al. [137] combined RNA and exomic DNA data with the Milan Criteria (an internationally accepted set of criteria for selecting patients with HCC for transplant) in a random forest model (AUC 0.90). However, these studies were limited by small sample sizes and tumour heterogeneity, limiting generalisability. Li et al. [138] used a gradient boosting survival model in 3,106 UNOS recipients (C-index 0.73), which outperformed the RETREAT score (C-index 0.70). Wu et al. [139] combined CT-based deep learning radiomics with clinical variables across two centres (validation AUC 0.83, rising to 0.86 when combined with the Metro-Ticket 2.0 criteria), and Ko et al. [140] applied a vision transformer to pathology images from 67 recipients to predict survival. Akbulut et al. [141] re-analysed a published cohort of 356 patients using nested cross-validation with all pre-processing confined to the training folds. Discrimination was moderate and similar across seven survival models, although all exceeded the Milan criteria; the authors attributed the near-perfect results of earlier re-analyses to information leakage between training and test data.

### Other long-term outcomes

Recent studies also addressed other long-term complications, all using single-centre data with internal validation only. Park et al. [142] found that machine learning models incorporating pre-transplant myocardial strain did not outperform penalised Cox regression or established risk scores for major adverse cardiovascular events (C-index 0.64, n = 309). Wang et al. [143] predicted refractory hypersplenism from pre-transplant CT and clinical data with a multimodal deep learning model (AUC 0.86, n = 164), and Zhang et al. [144] predicted biliary complications with LightGBM (validation AUC 0.75 compared with 0.70 for logistic regression, n = 133). Kim et al. [145] used generative AI to identify latent weight trajectories in 562 recipients, with MASH cirrhosis and male sex predicting later weight gain.

## Natural language processing

Four studies made use of natural language processing (NLP) in relation to liver transplantation. NLP is a branch of AI which attempts to understand and quantize human language.

Ge [146] (preprint) and Raz Bhutto’s [21] studies both used NLP to extract information from unstructured free text in EHRs. Ge et al. used a large language model (Versa Chat) to extract concepts from free text imaging reports of patients undergoing transplant for HCC. They found a variable degree of accuracy, ranging from 72.5% for tumour dimensions, to 99% for identifying macrovascular invasion in the imaging report. Raz Bhutto and colleagues applied a recurrent neural network to 1,380 notes of liver transplant recipients to map text to ICD-9 codes. This achieved a modest F1-score of 64.4%.

Dickerson et al. [147] used NLP on patient submitted messages to her institution. They assessed the difference in messages between patients with a MELD score of >30 versus < 30 in liver transplant recipients. They found that patients with a MELD score of >30 used shorter words and longer sentences, which they inferred may be an early marker of cognitive decline from metabolic encephalopathy (altered mental state secondary to the end-stage effects of liver disease). The latter study was small, with only 81 patients at a single centre, limiting generalisability, but demonstrates an innovative use of clinical NLP. Robitschek et al. [22] used a large language model framework to extract psychosocial risk factors and social determinants of health for transplant candidates. Each factor was associated with a 5–14 percentage point reduction in the probability of listing, and together these factors explained 42.6% of the racial disparity in listing decisions for Asian patients. As this study addresses the transplant evaluation decision, it is also linked to the pre-transplant stage in Fig 5.

## Discussion

This article presents a comprehensive overview of the literature on AI applications at every stage of the liver transplantation process. To our knowledge this is the largest review conducted to date on this topic, and is timely given the proliferation of AI applications in healthcare over the last 3 years.

We noted applications of AI to solving numerous problems in liver transplantation, ranging from prediction of survival in pre-transplant patients with end-stage liver disease, to more niche topics such as predicting the risk of pressure injury in children undergoing liver transplantation.

There were a variety of AI algorithms used, including established methods such as random forest, gradient boosting and traditional artificial neural networks for classification and regression tasks, but also NLP and computer vision models.

### Evidence gaps

A summary of the evidence gaps identified is shown in Table 1. Notably, despite the proliferation of large language models – based on the transformer architecture which has dominated AI research in recent years – there were few articles which made use of these. These models can be used for NLP as well as classification and regression tasks. This is despite the majority of articles being published after the first description of the transformer architecture in 2017 [148] and the release of ChatGPT 3 in 2022 which popularized AI [149,150]. To our knowledge, the TabPFN model [151], – based on a transformer architecture and optimised for tabular datasets with less than 10,000 observations – is the current state-of-the-art classification model [152,153]. In general more modern algorithms, such as LightGBM and CatBoost are sparsely evaluated despite being available prior to many of the studies included.

**Table 1:** Evidence gaps identified in scoping review.

Evidence Gap	Description
Poor quality training data	Majority of models developed on single centre retrospective data or registry data; limits generalisability of data and granularity of data available for model development
Lack of external validation	Minority of studies tested models on external datasets, limiting generalisability
Model drift	The performance of models may degrade over time as patient population and standards of care change, long-term evaluations of AI not available
Data harmonisation	Need for common data models (OMOP, FHIR) to allow training and testing of models across centres
Limited use of state of the art models	Few studies use modern LLMs, transformers and novel classifiers, despite recent advances
Explainability	Classical models preferred for interpretability, limited use of tools such as SHAP to derive variable importance
Early development stage	Only one model tested in a prospective trial, limited evidence of practical implementation of AI tools and impact on clinical decision-making once accuracy established

Despite the impressive performance metrics of many AI algorithms, they have still not displaced models based on classical statistical techniques such as logistic regression. As we see in this review, this is often because the AI models only marginally improve upon the performance of the classical models. Classical models have the advantage of being more explainable and intuitive to understand for clinicians, with the importance of a predictor variable being easy to identify with regression models able to produce familiar outcome measures such as odds and hazard ratios. They are also computationally more efficient; hence they have been preferred despite gains in accuracy with AI models.

While some papers here have attempted to clarify the importance of variables using methods such as Shapley Additive Explanations (SHAP), this produces importance weights that do not translate easily into clinical effectiveness measurements such as odds or hazard ratios. Several models also use an inordinate number of predictor variables, in some cases >200, limiting utility, explainability and may result in overfitting of the model on the data. Further, there are a far larger number of parameters, such as the number of layers and weights in a neural network, learning rate and batch size than are typically needed in classical regression models. The characteristics of the training dataset, the level of missing data within and the method of training and test sample split, with any resampling methods such as k-fold cross-validation are also significant in producing a model [154]. In our included papers, these factors were seldom described in sufficient detail to allow reproduction of the AI-based models. These issues are also present in traditional statistical models [155], however, the added complexity of AI models may hinder trust in them with many being considered a ‘black box [156].’

The data being used to develop most of the models in this review (n = 105, 53.6%) consist of single-centre, retrospective data. While some of these datasets are large, with several thousand patients, the single-centre samples will limit external validity, and few studies attempted external validation (n = 27, 13.8%). Most multi-centre datasets are also from established national transplant registries such as the UNOS dataset in the United States. Models developed on these have been shown to have limited applicability in other national registries as seen in the study by Ivanics [95]. This may be because of different patient populations and clinical management in different countries, but issues around harmonization of data fields and differing levels of missing data, led to many variables being excluded to allow application of their model to international datasets. Developing datasets using common data models such as the Observational Medical Outcomes Partnership (OMOP [157]) and Fast Healthcare Interoperability Resources (FHIR, [158]) with structured vocabularies such as SNOMED-CT [159] for documenting values, may mitigate this and allow interoperability between datasets.

### Ethical, governance, and workflow integration challenges

Beyond data granularity and performance metrics, transitioning AI tools from *in silico* validation to *in vivo* clinical transplantation workflows presents profound ethical, regulatory and practical hurdles:

- **Fairness and Algorithmic Bias:** A primary ethical concern in liver allocation is ensuring that models do not inadvertently perpetuate or amplify existing healthcare disparities. AI algorithms learn entirely from historical data. If the training datasets reflect underlying structural inequities- such as historical disparities in waitlist access or post-transplant follow-up based on gender, race, geography, or socioeconomic status- the model risks encoding these biases as objective allocation rules. For instance, models optimized purely for post-transplant survival metrics might systematically deprioritize clinically marginal or vulnerable patient populations who possess fewer post-operative social support structures, challenging the fundamental ethical principle of equity in organ stewardship. Such bias has been demonstrated in practice, with a widely used US algorithm substantially underestimating illness severity in Black patients [160]. Unrepresentative data compound this risk: over half of the models in this review were developed on single-centre data, and over half of all studies (n = 108, 55.1%) used data from the USA or China.
- **Explainability and Liability:** The “black box” nature of advanced deep learning and transformer models introduces complex professional and legal issues. Under current medical governance frameworks, clinicians bear final accountability for treatment decisions. If a neural network recommends discarding a donor graft or denying waitlist access, a clinician cannot ethically or legally accept that recommendation without understanding its underlying clinical rationale. Post-hoc explainability frameworks like SHAP provide variable importance weights, but they do not replicate clinical reasoning. Despite an awareness of such limitations, it is established that clinicians are subject to automation bias and may accept recommendations from an AI model as the default [161], which may be harmful given a flawed model. Liability for AI-related errors, shared between clinicians, institutions and developers, also remains legally untested [162].
- Data Protection and Consent: Model development requires large volumes of sensitive health data, and supposedly anonymised records can often be re-identified [163]. Health data are also vulnerable to attack, as shown by the 2024 ransomware attack on pathology services at King’s College Hospital and Guy’s and St Thomas’ in the UK, which postponed thousands of appointments and procedures [164]. Patients also wish to be told when AI is used in their care [165]; transparent consent is particularly important in transplantation, where public trust underpins organ donation.
- **Regulatory Hurdles:** Regulatory bodies (such as the FDA [166] or MHRA [167] in the USA and UK, respectively) increasingly mandate that clinical AI is regulated in a manner similar to physical medical devices such as prostheses. This concept is known as “Software as a Medical Device” (SaMD) with its own set of regulatory standards for development and post-market surveillance protocols [168].
- **Integration into Clinical Workflows:** Even an algorithm with perfect predictive accuracy will fail to improve patient outcomes if it cannot be integrated into high-stress, time-critical clinical workflows. Organ retrieval and allocation operate on compressed timelines, often over a matter of hours overnight. If a computer vision tool for graft assessment requires manual data cleaning, specialized local hardware, or lengthy computing times, it cannot be utilized at the donor bedside.
- Environmental Sustainability: Training and running large AI models consumes substantial energy; training a single large model can emit hundreds of tonnes of CO2 [169]. ‘Green AI’ approaches, which report and minimise computational cost, favour smaller models where performance is comparable [170]. As most models in this review offered only marginal gains over logistic regression, the energy cost of more complex models should be justified by clear clinical benefit.
- **Model drift** occurs when the characteristics of the population being predicted and standard of care change such that the model no longer makes accurate predictions- mitigation requires continuous evaluation of a model in practice to ensure consistent accuracy
- Even if the above are all solved, even with perfect accuracy, clinicians, patients and health systems may still not alter their established practices- prospective evaluations of AI are needed to validate their benefit

Nearly all the models in this paper are at early stages of development (IDEAL Stage 0, as they are all being evaluated in silico rather than being used clinically). When treating AI as a medical device, only one study was prospective and reached stage 3 of the IDEAL framework [171]. This was the recently published study by Khong and colleagues [124], which evaluated the clinical impact of their AI model for predicting tacrolimus dosing with the standard of care in a randomized controlled trial. This has highlighted the potential of AI, with the model outperforming standard of care dosing. Ongoing trials such as the national OrQA trial in the UK using computer vision techniques to assess graft quality [172], show great promise in driving forward the use of AI in transplantation.

### Recommendations for future research

High-quality data, harmonized to common data models with clear data dictionaries are necessary in liver transplant datasets to allow development of high-quality AI models and allow easy external validation of these models.

Development of AI should be transparent with all model parameters and training processes published to allow reproducibility. Predictor variables should be explainable clinically and quantitatively with clear descriptions of their importance to their models, further, the number of predictors should be minimised to aid utility and explainability.

The number of decisions and outcomes in liver transplantation is large and is unlikely to be predicted by a single model. However, a single model focusing on, for example, quantifying the risk of various post-operative outcomes at different time-points may simplify risk prediction for patients and clinicians.

## Supporting information

S1 File PRISMA-ScR Checklist. (DOCX)

S2 File Search terms for Embase and MEDLINE. (DOCX)

S3 File Reference list of included articles in review. (XLSX)

S4 File Bibliometric analysis report. (XLSX)
