# Supplementary material for: The applications of artificial intelligence in liver transplantation: A comprehensive scoping review
Source: PLoS One. 2026 Oct 23;21(10):e0361120. doi: 10.1371/journal.pone.0361120 (PMC13693877; doi:10.1371/journal.pone.0361120)
Supplement: Supplementary file 2 [file pone.0361120.s002.docx]

Ovid MEDLINE(R) ALL <1946 to September 25, 2026>

1	((liver or hepatic) and (transplant* or graft) and ("natural language processing" or NLP or "big data")).mp. [mp=title, book title, abstract, original title, name of substance word, subject heading word, floating sub-heading word, keyword heading word, organism supplementary concept word, protocol supplementary concept word, rare disease supplementary concept word, unique identifier, synonyms, population supplementary concept word, anatomy supplementary concept word]	36

2	((liver or hepatic) and (transplant* or graft) and ("natural language processing" or NLP or "big data" or AI or artificial intelligence or machine learning or neural or large language model or LLM)).mp. [mp=title, book title, abstract, original title, name of substance word, subject heading word, floating sub-heading word, keyword heading word, organism supplementary concept word, protocol supplementary concept word, rare disease supplementary concept word, unique identifier, synonyms, population supplementary concept word, anatomy supplementary concept word]	826

3	exp Liver Transplantation/	67982

4	exp Artificial Intelligence/	239929

5	exp Natural Language Processing/	8058

6	exp Large Language Models/	406

7	4 or 5 or 6	239929

8	3 and 7	171

9	2 or 8	859

10	limit 9 to yr="2000 -Current"	768

Embase <1974 to 2026 September 25>

1	((liver or hepatic) and (transplant* or graft) and ("natural language processing" or NLP or "big data" or AI or artificial intelligence or machine learning or neural network or large language model or LLM)).mp. [mp=title, abstract, heading word, drug trade name, original title, device manufacturer, drug manufacturer, device trade name, keyword heading word, floating subheading word, candidate term word]	1493

2	exp liver graft/	35906

3	exp natural language processing/	16081

4	exp artificial intelligence/	139699

5	exp machine learning/	584531

6	exp artificial neural network/	133468

7	exp large language model/	9312

8	3 or 4 or 5 or 6 or 7	650285

9	2 and 8	249

10	1 or 9	1554

11	limit 10 to yr="2000 -Current"	1526

12	limit 11 to ("remove clinical trial (clinicaltrials.gov) records" and "remove medline records")	813
