# Supplementary material for: The applications of artificial intelligence in liver transplantation: A comprehensive scoping review
Source: PLoS One. 2026 Oct 23;21(10):e0361120. doi: 10.1371/journal.pone.0361120 (PMC13693877; doi:10.1371/journal.pone.0361120)
Supplement: Supplementary file 4 [file pone.0361120.s007.docx]

“Aditya Borakati is supported by the UK Engineering and Physical Sciences Research Council (EPSRC) [Grant reference number EP/Y035216/1] Centre for Doctoral Training in Data-Driven Health (DRIVE-Health) at King's College London, with additional funding from the Roger Williams Institute of Liver Studies at King’s College London and a Fellowship in Clinical Artificial Intelligence from the National Health Service Digital Academy.”

Please state what role the funders took in the study.  If the funders had no role, please state: "The funders had no role in study design, data collection and analysis, decision to publish, or preparation of the manuscript."

If this statement is not correct you must amend it as needed.

Please include this amended Role of Funder statement in your cover letter; we will change the online submission form on your behalf.

*Thank you. This information has been added to the cover letter.*

3. Please provide a complete Data Availability Statement in the submission form, ensuring you include all necessary access information or a reason for why you are unable to make your data freely accessible. If your research concerns only data provided within your submission, please write "All data are in the manuscript and/or supporting information files" as your Data Availability Statement.

*Thank you. This information has now been added.*

4. We note that Figure 4 in your submission contain map images which may be copyrighted. All PLOS content is published under the Creative Commons Attribution License (CC BY 4.0), which means that the manuscript, images, and Supporting Information files will be freely available online, and any third party is permitted to access, download, copy, distribute, and use these materials in any way, even commercially, with proper attribution. For these reasons, we cannot publish previously copyrighted maps or satellite images created using proprietary data, such as Google software (Google Maps, Street View, and Earth). For more information, see our copyright guidelines: http://journals.plos.org/plosone/s/licenses-and-copyright.

We require you to either (1) present written permission from the copyright holder to publish these figures specifically under the CC BY 4.0 license, or (2) remove the figures from your submission:

1. You may seek permission from the original copyright holder of Figure(s) [#] to publish the content specifically under the CC BY 4.0 license.

We recommend that you contact the original copyright holder with the Content Permission Form (http://journals.plos.org/plosone/s/file?id=7c09/content-permission-form.pdf) and the following text:

“I request permission for the open-access journal PLOS ONE to publish XXX under the Creative Commons Attribution License (CCAL) CC BY 4.0 (http://creativecommons.org/licenses/by/4.0/). Please be aware that this license allows unrestricted use and distribution, even commercially, by third parties. Please reply and provide explicit written permission to publish XXX under a CC BY license and complete the attached form.”

Please upload the completed Content Permission Form or other proof of granted permissions as an "Other" file with your submission.

In the figure caption of the copyrighted figure, please include the following text: “Reprinted from [ref] under a CC BY license, with permission from [name of publisher], original copyright [original copyright year].”

*I can confirm that this is a computer-generated file using the open source plotly library, generated by myself, therefore the copyright belongs to me. I am happy to grant permission to use this file by PLOS ONE under the CC BY 4.0 license as the copyright holder.*

2. If you are unable to obtain permission from the original copyright holder to publish these figures under the CC BY 4.0 license or if the copyright holder’s requirements are incompatible with the CC BY 4.0 license, please either i) remove the figure or ii) supply a replacement figure that complies with the CC BY 4.0 license. Please check copyright information on all replacement figures and update the figure caption with source information. If applicable, please specify in the figure caption text when a figure is similar but not identical to the original image and is therefore for illustrative purposes only.

The following resources for replacing copyrighted map figures may be helpful:

USGS National Map Viewer (public domain): http://viewer.nationalmap.gov/viewer/

The Gateway to Astronaut Photography of Earth (public domain): http://eol.jsc.nasa.gov/sseop/clickmap/

Maps at the CIA (public domain): https://www.cia.gov/library/publications/the-world-factbook/index.html and https://www.cia.gov/library/publications/cia-maps-publications/index.html

NASA Earth Observatory (public domain): http://earthobservatory.nasa.gov/

Landsat: http://landsat.visibleearth.nasa.gov/

USGS EROS (Earth Resources Observatory and Science (EROS) Center) (public domain): http://eros.usgs.gov/#

Natural Earth (public domain): http://www.naturalearthdata.com/

*N/A*

5. We note you have included a table to which you do not refer in the text of your manuscript. Please ensure that you refer to Table 1 in your text; if accepted, production will need this reference to link the reader to the Table.

*Thank you, This table is now referenced in line 502.*

6. If the reviewer comments include a recommendation to cite specific previously published works, please review and evaluate these publications to determine whether they are relevant and should be cited. There is no requirement to cite these works unless the editor has indicated otherwise.

# Reviewers' comments: Reviewer's Responses to Questions

**Comments to the Author**

1. Is the manuscript technically sound, and do the data support the conclusions?

The manuscript must describe a technically sound piece of scientific research with data that supports the conclusions. Experiments must have been conducted rigorously, with appropriate controls, replication, and sample sizes. The conclusions must be drawn appropriately based on the data presented.

Reviewer #1: Yes

Reviewer #2: No

2. Has the statistical analysis been performed appropriately and rigorously?

Reviewer #1: No

Reviewer #2: N/A

3. Have the authors made all data underlying the findings in their manuscript fully available?

The [PLOS Data policy](http://track.editorialmanager.com/CL0/http:%2F%2Fwww.plosone.org%2Fstatic%2Fpolicies.action%23sharing/1/010f019e5d11e5e8-bafb471a-9755-4ffb-95c9-9dc36ce024a1-000000/Lfoiq1QQSHYaYR-cZE6Gf4SCHNsmsKkrUaVRPaQSGMM=258) requires authors to make all data underlying the findings described in their manuscript fully available without restriction, with rare exception (please refer to the Data Availability Statement in the manuscript PDF file). The data should be provided as part of the manuscript or its supporting information, or deposited to a public repository. For example, in addition to summary statistics, the data points behind means, medians and variance measures should be available. If there are restrictions on publicly sharing data—e.g. participant privacy or use of data from a third party—those must be specified.

Reviewer #1: Yes

Reviewer #2: Yes

4. Is the manuscript presented in an intelligible fashion and written in standard English?

PLOS ONE does not copyedit accepted manuscripts, so the language in submitted articles must be clear, correct, and unambiguous. Any typographical or grammatical errors should be corrected at revision, so please note any specific errors here.

Reviewer #1: No

Reviewer #2: Yes

5. Review Comments to the Author

Please use the space provided to explain your answers to the questions above. You may also include additional comments for the author, including concerns about dual publication, research ethics, or publication ethics. (Please upload your review as an attachment if it exceeds 20,000 characters)

Reviewer #1: The authors have conducted an in-depth review of the application of AI in liver transplantation across the different stages of the process. However, the way in which the data are presented could be improved. Incorporating a bibliometric analysis, as well as reporting table-level metrics such as accuracy, sensitivity, and specificity to identify the most effective methods at each stage of the liver transplant process, would help guide readers in determining the most robust approaches described in the literature.

*Many thanks for your comments.*

*At the time of submission we had included a bar chart showing included papers by year and a heat map by country (figures 1 and 2). In direct response to your suggestions, we have now conducted a full bibliometric analysis (methods lines 198-202) and left the full report from our software as a supplementary file whilst highlighting key findings in the main body (lines 220-224 and S4).*

*With regard to reporting additional metrics such as sensitivity, specificity etc., we deliberately chose to focus on the key summary statistics as we feel it would make the paper far too large. The study types, patient populations, purposes of the models and reported accuracy metrics are vastly different, therefore aggregating such metrics even in a table may be misleading and inappropriate. We greatly appreciate the rationale underlying reviewer’s comments, but as a scoping review we only seek to provide an overview of the themes in the literature.*

Additionally, the authors provide interesting comments on SHAP models in the discussion section; however, only a few papers included these visualizations in the review. It would be helpful to begin the paper with a basic overview of how AI training models have evolved, and to emphasize the importance of model interpretability description. This would likely make the manuscript more readable and accessible.

*Many thanks, we agree with these comments and have rewritten and expanded the introduction to provide this overview. (pages 6-8, lines 107-158)*

Minor comments
Please review the sentences on page 9 (lines 184–187) and page 24 (line 504), as they appear to have been included in the text by mistake.

*Thank you for highlighting this issue. These seem to be errors originating from the conversion to PDF in the editorial system, they do not appear in the Word document version which we have uploaded. On resubmission we will as much as possible endeavour to make sure the same issue does not arise again.*

Reviewer #2: This manuscript addresses a highly relevant and timely topic, providing a comprehensive overview of artificial intelligence applications across the liver transplantation pathway. The review is well structured and likely represents one of the largest scoping reviews currently available in this field. However, several methodological and conceptual issues should be addressed before publication.

*Many thanks for your review and kind comments. We will attempt to address as many of these issues as we possibly can.*

1. A major limitation is the overly broad definition of “artificial intelligence,” as the manuscript groups together highly heterogeneous approaches including logistic regression-based models, machine learning classifiers, deep learning, NLP, and transformers without a clear conceptual distinction. Greater methodological stratification would improve clarity and interpretation.

*Many thanks for this comment, we agree and we have added and rewritten the introduction to provide an overview of AI concepts and better define these (pages 6-8, lines 107-158). As an example, we have chosen to group regression with machine learning as many machine learning packages still contain automated methods of incorporating regression with techniques such as LASSO. We feel this is distinct from the manual and traditional way statisticians typically fit regression models.*

2. The review also lacks a formal assessment of methodological quality or risk of bias. Many included studies are small retrospective single-centre cohorts with limited external validation and possible overfitting, despite reporting very high performance metrics. Incorporating a structured critical appraisal framework would strengthen the manuscript considerably.

*Many thanks for this feedback, we did consider this, however, the PRSIMA-ScR and other scoping review guidelines do not recommend a traditional risk of bias assessment as in a traditional systematic review. Further, we note similar scoping reviews in this journal without such assessments:* [*https://journals.plos.org/plosone/article?id=10.1371/journal.pone.0260471*](https://journals.plos.org/plosone/article?id=10.1371/journal.pone.0260471)*.*

*As this review has selected a large number of articles with multiple different and heterogeneous study designs, we would need to use a range of different risk of bias assessment tools which would not be possible to aggregate. We therefore feel that it would not be necessary or helpful to include a risk of bias assessment in this paper. We have now explained our rationale in the methods (lines 192-194)*

3. Although the discussion appropriately highlights limitations such as poor external validation and retrospective datasets, the Results section occasionally presents model performance too positively without sufficient scepticism regarding reproducibility or clinical applicability. In several examples, the clinical utility of modest improvements in predictive accuracy remains unclear.

*Many thanks, this is an important point and thank you for noting it. We have added additional critiques of the results in each domain. We did have some discussion of the modest improvements, being insufficient to displace simpler statistical models in the results, but have expanded upon this in the Discussion.*

4. The manuscript would also benefit from a more detailed discussion of ethical and regulatory aspects of clinical AI implementation, including explainability, fairness, governance, and integration into real-world transplant workflows.

*Thank you, this is also an important point – we have added a section covering these issues to the Discussion (lines 638-680).*

5. Several editorial issues should also be corrected, including unresolved reference errors (“Error! Reference source not found”). In addition, terminology is occasionally inconsistent, with AI, machine learning, and deep learning sometimes used interchangeably.

*Many thanks, this appears to be an error with the journal’s editorial manager converting the Word document to a PDF; this should now be fixed. We have clarified the terminology in the Introduction in our new section (pages 6-8)*

6. The inclusion of preprint studies from platforms such as medRxiv and arXiv without a separate sensitivity or subgroup analysis represents an important limitation of the review. Since these studies have not undergone peer review, their methodological quality and reliability may be variable. The authors should clarify the proportion of included preprints and discuss whether their inclusion could have influenced the overall conclusions of the manuscript.

*Many thanks, we have included 4 articles from preprint servers and in response to your comments we have now signposted which articles are preprints within the text (lines 259, 274, 554) As this is not a meta-analysis and is only a scoping review with a narrative description, it is unfortunately not possible to perform a sensitivity analysis or subgroup analysis. Again, in response to your comments we re-reviewed all included pre-prints and feel that methodologically they are of the same quality as the published articles and in the interests of consistency have described them in the same way in our narrative albeit in doing so have acknowledged that these particular articles have not been subjected to peer review.*

7. The manuscript would benefit from the inclusion of a summary table or schematic figure clearly illustrating the current major applications of artificial intelligence in liver transplantation. A visual overview categorising AI applications across the transplant pathway (pre-transplant assessment and allocation, graft evaluation, intraoperative support, short-term complications, long-term outcomes, immunosuppression management, and NLP applications) would improve readability and help readers better contextualise the breadth and maturity of the field.

*Thank you for this comment. We definitely agree and as such provided this information in this format in figure 5. We have reviewed this figure again and believe it displays the applications we have identified in the literature and partitions them within the table in an almost identical structure as you have suggested.*

8. Overall, this is an important and valuable review with strong potential impact, but it would benefit from clearer methodological definitions, more critical appraisal of included studies, and stronger clinical contextualisation before publication.

*Many thanks once again for your thorough and considered review. We have worked hard to adapt the manuscript in accordance with the suggestions provided.*

6. PLOS authors have the option to publish the peer review history of their article ([what does this mean?](https://track.editorialmanager.com/CL0/https:%2F%2Fjournals.plos.org%2Fplosone%2Fs%2Feditorial-and-peer-review-process%23loc-peer-review-history/1/010f019e5d11e5e8-bafb471a-9755-4ffb-95c9-9dc36ce024a1-000000/lpctjvR1NSEOUF0SX_iaw1e4EMO-FU4QDbIWqRmwl5k=258)). If published, this will include your full peer review and any attached files.


If you choose “no”, your identity will remain anonymous but your review may still be made public.


**Do you want your identity to be public for this peer review?** For information about this choice, including consent withdrawal, please see our [Privacy Policy](https://track.editorialmanager.com/CL0/https:%2F%2Fwww.plos.org%2Fprivacy-policy/1/010f019e5d11e5e8-bafb471a-9755-4ffb-95c9-9dc36ce024a1-000000/4piNVeYroixtq1z2uLNdmkfBg3DAqokFZzCIC3f7drk=258).

Reviewer #1: No

Reviewer #2: No

[NOTE: If reviewer comments were submitted as an attachment file, they will be attached to this email and accessible via the submission site. Please log into your account, locate the manuscript record, and check for the action link "View Attachments". If this link does not appear, there are no attachment files.]
